# Antidepressants are an Important Part of Treating Depression

**DOI:** 10.3389/fpsyg.2012.00240

**Published:** 2012-07-20

**Authors:** Jitender Sareen, Murray W. Enns

**Affiliations:** ^1^Department of Psychiatry, University of ManitobaWinnipeg, MB, Canada; ^2^Department of Psychology, University of ManitobaWinnipeg, MB, Canada; ^3^Department of Community Health Sciences, University of ManitobaWinnipeg, MB, Canada; ^4^Canadian Psychiatric AssociationOttawa, ON, Canada

A recent review paper, published online on April 24 2012 in Frontiers in Psychology, argues that antidepressants interfere with the normal homeostatic response of the body and cause more harm than good (Andrews et al., 2012). The authors cite a number of studies that show that antidepressants are associated with a range of side effects, that drug-placebo differences are small for mild to moderate depression, and that people on antidepressants may die prematurely.

Although we fully agree that the benefits of an intervention should outweigh the risks, the authors have presented a biased analysis of the literature. Their conclusion that antidepressants are more harmful than helpful is unwarranted. Media reporting of this biased conclusion could be harmful to patients struggling with depression who are trying to make difficult decisions related to treatment.

Depression is a mental illness that affects individuals of all ages, and across Canada. Rates of acute hospitalization for depression are consistently higher than for any other mental illness (Canadian Institute for Health Information, 2008). And as a predictor of early death, depression is on a par with smoking (Mykletun, 2009). Worldwide, depression is the leading cause of years lived with disability (Ustun et al., 2004). It can disrupt many aspects of life, such as maintaining employment and/or productivity in the workplace (Marcotte and Wilcox-Gok, 2001; Lerner et al., 2004; Virtanen et al., 2005). In fact, the negative impact of depression on job performance has been estimated to be greater than that of chronic conditions such as arthritis, hypertension, back problems, and diabetes (Wells et al., 1989; Kessler et al., 2001).

There are current standards on minimizing the bias of reviewing the literature (Stroup et al., 2000). However, the authors do not provide any information on how they selected the articles cited and only cite articles that support their argument.

Furthermore, a number of important studies that counter the authors’ arguments are not included in their review. For example, ecological studies have shown that increases in the use of antidepressants are associated with lower rates of suicide (Olfson et al., 2003). A Manitoba study demonstrated that after the Health Canada warnings, the resulting reduction in the prescription of antidepressants was associated with an increase in youth suicides (Katz et al., 2008). A number of studies have demonstrated that the risk of suicide attempts is highest *before* the initiation of an antidepressant (Gibbons et al., 2005, 2007). A recent study by Gibbons et al. (2012) demonstrates that antidepressants decrease the rate of suicidal ideation and suicide attempts.

The authors of the review in Frontiers in Psychology argue that antidepressants interfere with the “natural homeostatic response.” They even argue that treating a fever with Tylenol can interfere with the normal homeostatic response to the body. Although we can appreciate some aspects of their argument, there are many conditions where interference with the body's “natural” response is needed. Many physical illnesses such as diabetes, hypertension, cardiovascular disease, rheumatologic diseases, cancer and allergies are associated with abnormalities in natural responses to environmental triggers. Do the authors suggest that we should not treat these illnesses? Some highly toxic side-effects are expected with treatments of cancer and other serious physical illnesses. However, the patient and health care provider have to weigh the risks and benefits of the intervention with the disability and outcomes associated with illness. As we have argued above, the distress, disability, and mortality associated with depressive illness is enormous on both an individual and societal scale.

Yes, evidence based psychotherapies and antidepressants are equally effective. Antidepressant medication treatment should always be combined with psychotherapy. If available, psychotherapy should be considered as first line treatment for mild to moderate depression. However, evidence based psychotherapies are not necessarily readily available and there is a need to advocate for greater numbers of health care providers that can provide these therapies. It is worthy of note that a recent New England Journal of Medicine study, also not cited by the authors, demonstrated that collaborative care treatment of depression (including antidepressants and nurse provided therapy) improve both mental and physical health outcomes (Katon et al., 2010).

Finally, the argument that evidence from randomized control trials that show little difference between antidepressants and placebos proves that antidepressants are not effective is a simplistic interpretation of the data. Recent work has demonstrated that these trials show small mean differences between the two groups because they lump responders together with non-responders. When looking at subgroups of responders, there are large differences between antidepressants and placebo (Thase et al., 2011).

## References

[B1] Andrews P. W. Thomson J. A. Jr. Amstadter A. Neale M. C. (2012). Primum non nocere: an evolutionary analysis of whether antidepressants do more harm than good. Front. Psychology 3:117 10.3389/fpsyg.2012.00117 22536191 PMC3334530

[B2] Canadian Institute for Health Information (2008). Hospital Mental Health Services in Canada, 2005–2006. Available at: http://www.cpa.ca/cpasite/userfiles/Documents/Practice_Page/Hmhdb_annual_report_2008_e.pdf

[B3] Gibbons R. D. Brown C. H. Hur K. Davis J. M. Mann J. J. (2012). Suicidal thoughts and behavior with antidepressant treatment: reanalysis of the randomized placebo-controlled studies of Fluoxetine and Venlafaxine. Arch. Gen. Psychiatry. [Epub ahead of print]. 22309973 10.1001/archgenpsychiatry.2011.2048 PMC3367101

[B4] Gibbons R. D. Brown C. H. Hur K. Marcus S. M. Bhaumik D. K. Mann J. J. (2007). Relationship between antidepressants and suicide attempts: an analysis of the Veterans Health Administration data sets. Am. J. Psychiatry 164, 1044–1049 10.1176/appi.ajp.2007.07091463r 17606656

[B5] Gibbons R. D. Hur K. Bhaumik D. K. Mann J. J. (2005). The relationship between antidepressant medication use and rate of suicide. Arch. Gen. Psychiatry 62, 165–172 10.1001/archpsyc.62.2.165 15699293

[B6] Katon W. J. Lin E. H. Von Korff M. Ciechanowski P. Ludman E. J. Young B. Peterson D. Rutter C. M. McGregor M. McCullough D. (2010). Collaborative care for patients with depression and chronic illnesses. N. Engl. J. Med. 363, 2611–2620 10.1056/NEJMoa1003955 21190455 PMC3312811

[B7] Katz L. Y. Kozyrskyj A. L. Prior H. Enns M. W. Cox B. J. Sareen J. (2008). Effect of regulatory warnings on antidepressant prescription rates, use of health services and outcomes among children and adolescents. CMAJ 178, 1–7 10.1503/cmaj.071265 PMC2276558 18390943

[B8] Kessler R. C. Greenberg P. E. Mickelson K. D. Meneades L. M. Wang P. S. (2001). The effects of chronic medical conditions on work loss and work cutback. J. Occup. Environ. Med. 43, 218–225 10.1097/00043764-200103000-00009 11285869

[B9] Lerner D. Adler D. A. Chang H. Lapitsky L. Hood M. Y. Permissinotto C. Reed J. McLaughlin T. J. Berndt E. R. Rogers W. H. (2004). Unemployment, job retention, and productivity loss among employees with depression. Psychiatr. Serv. 55, 1371–1378 10.1176/appi.ps.55.12.1371 15572564 PMC4283817

[B10] Marcotte D. E. Wilcox-Gok V. (2001). Estimating the employment and earnings costs of mental illness: recent developments in the United States. Soc. Sci. Med. 53, 21–27 10.1016/S0277-9536(00)00312-9 11386306

[B11] Mykletun A. (2009). Levels of anxiety and depression as predictors of mortality: the HUNT study. Br. J. Psychiatry 195, 118–125 10.1192/bjp.bp.108.054866 19648541

[B12] Olfson M. Shaffer D. Marcus S. C. Greenberg T. (2003). Relationship between antidepressant medication treatment and suicide in adolescents. Arch. Gen. Psychiatry 60, 978–982 10.1001/archpsyc.60.9.978 14557142

[B13] Stroup D. F. Berlin J. A. Morton S. C. Olkin I. Williamson G. D. Rennie D. Moher D. Becker B. J. Stipe T. A. Thacker S. B. (2000). Meta-analysis of observational studies in epidemiology: a proposal for reporting. JAMA 283, 2008–2012 10.1001/jama.283.15.2008 10789670

[B14] Thase M. E. Larsen K. G. Kennedy S. H. (2011). Assessing the true effect of active antidepressant therapy versus placebo in major depressive disorder: use of a mixture model. Br. J. Psychiatry 199, 501–507 10.1192/bjp.bp.111.093336 22130749

[B15] Ustun T. B. Yuso-Mateos J. L. Chatterji S. Mathers C. Murray C. J. L. (2004). Global burden of depressive disorders in the year 2000. Br. J. Psychiatry 184, 386–392 10.1192/bjp.184.5.386 15123501

[B16] Virtanen M. Kivimaki M. Elovainio M. Vahtera J. Kokko K. Pulkkinen L. (2005). Mental health and hostility as predictors of temporary employment: evidence from two prospective studies. Soc. Sci. Med. 61, 2084–2095 10.1016/j.socscimed.2005.04.028 15941612

[B17] Wells K. B. Stewart A. Hays R. D. Burnam M. A. Rogers W. Daniels M. Berry S. Greenfield S. Ware J. (1989). The functioning and well-being of depressed patients: results from the Medical Outcomes Study. JAMA 262, 914–919 10.1001/jama.262.23.3298 2754791

